# Supplementary material for: A new growing degree-day phenology model for wheat stem sawfly (Hymenoptera: Cephidae) in Colorado wheat fields
Source: PLoS One. 2025 Apr 7;20(4):e0320497. doi: 10.1371/journal.pone.0320497 (PMC11975124; doi:10.1371/journal.pone.0320497)
Supplement: S1 Table — (DOCX) [file pone.0320497.s001.docx]

**S1 Table. Summary of average temperature, rainfall, snowfall, and wheat stem sawfly counts per year.**

| Year | Avg Temperature (°C) | Avg Rainfall (mm) | Avg Snowfall (mm) | Avg WSS Count | Avg WSS Count (Log) |
| --- | --- | --- | --- | --- | --- |
| 2011 | 1.89 | 1.11 | 3.87 | 115 | 4.74 |
| 2012 | 4.89 | 0.71 | 2.15 | 340 | 5.83 |
| 2013 | 1.88 | 0.94 | 7.90 | 65 | 4.17 |
| 2014 | 1.92 | 1.05 | 7.82 | 110 | 4.70 |
| 2016 | 3.53 | 1.31 | 6.81 | 325 | 5.78 |
| 2017 | 4.15 | 1.44 | 2.40 | 300 | 5.70 |
| 2018 | 2.81 | 1.43 | 5.81 | 360 | 5.89 |
| 2019 | 1.09 | 1.24 | 5.73 | 200 | 5.30 |
| 2020 | 2.81 | 0.64 | 4.27 | 415 | 6.03 |
| 2021 | 1.89 | 1.10 | 5.16 | 285 | 5.65 |
| 2022 | 2.25 | 0.49 | 4.04 | 120 | 4.79 |
| 2023 | 0.84 | 1.48 | 7.50 | 90 | 4.50 |
