# Supplementary material for: A new growing degree-day phenology model for wheat stem sawfly (Hymenoptera: Cephidae) in Colorado wheat fields
Source: PLoS One. 2025 Apr 7;20(4):e0320497. doi: 10.1371/journal.pone.0320497 (PMC11975124; doi:10.1371/journal.pone.0320497)
Supplement: Attachment — Submitted filename: Vieira et al. PLOSONE_Final - updated.docx [file pone.0320497.s002.docx]

**A new degree-day phenology model for wheat stem sawfly (Hymenoptera: Cephidae) in Colorado wheat fields**

Henrique V. Vieira^1^, Benjamin Z. Bradford^2^, Adam Osterholzer,^1,^ Erika S. Peirce^4^, Darren Cockrell^5^, Frank Peairs^1^, Kenneth Frost^3^, Russell Groves^2^, and Punya Nachappa^1*^

^1^Department of Agricultural Biology, Colorado State University, Fort Collins, Colorado, USA

^2^Department of Entomology, University of Wisconsin-Madison, Madison, Wisconsin, USA

^3^Hermiston Ag Research and Extension Center, Hermiston, Oregon, United States of America

^4^ Rangeland Resources and SystemsResearch Unit, USDA-ARS, Fort Collins, CO,USA

^5^ School of Global Environmental Sustainability, Colorado State University, Fort Collins, Colorado, USA

*Corresponding author, e-mail: [punya.nachappa@colostate.edu](mailto:punya.nachappa@colostate.edu)

# Abstract

Wheat stem sawfly (WSS), *Cephus cinctus*(Hymenoptera: Cephidae), is a native grass-feeding insect and one of the most important pests of wheat in North America. Yield losses from WSS can be due to stem boring and/or stem cutting which causes plants to lodge.Current methods, such as solid stem varieties and insecticides, do not effectively control WSS. A better understanding of WSS emergence, population size, and related environmental factors is critical to building efficient and effective integrated pest management (IPM) strategies for this pest. In this study, wheat fields were sampled for adult WSS from mid-April to the end of June between 2011 and 2023 in several field sites in two locations in Colorado. This multi-year data created a phenology model that predicts adult WSS emergence and population peak based on growing DD (GDD). The inter-annual variability in emergence timing based on calendar date was substantially reduced when using a GDD model with a base temperature of 10°C, an upper threshold of 30°C, and a biofix of Jan 1. The model predicted initial WSS emergence at 148 DD, population peak at 224 DD, and decline at 354 DD. We also modeled the effects of environmental factors on mean WSS populations at each field site, finding that higher WSS populations are associated with longer emergence periods, less precipitation before emergence, milder temperatures during emergence, and milder maximum temperatures before and during emergence. By analyzing multiple years of comprehensive phenology data, we provide robust models to guide adult WSS forecasting and monitoring for the first time. Further, this data will aid in decision-making related to timely and effective management strategies to suppress populations of WSS.

# Introduction

The wheat stem sawfly (WSS), *Cephus cinctus* Norton(Hymenoptera: Cephidae) is a native grass-feeding insect and is considered to be one of the most economically important pests of wheat in North America[1, 2]. The plant injury is mainly caused by stem boring and/or stem cutting by WSS larvae. In addition, WSS can reduce photosynthetic activity[3], cause grain weight reduction [4, 5], and cause significant yield loss by toppling stems[6]**,** generating difficulties in harvesting. WSS has traditionally infested spring wheat, but over the last few decades, the damage has become increasingly common in winter wheat[2, 7]. WSS damage has been estimated at up to 350 million dollars per year in the northern Great Plains region[1], including $41 million in damage in 2022 to Colorado winter wheat production[8].

WSS is univoltine with adult emergence from overwintering pupae influenced by temperature[9, 10].Diapause requires approximately 90 days below 10°C, with many individuals remaining in or re-entering diapause at temperatures of 10°C or below and 35°C or above[10, 11].In the spring, WSS completes diapause as a final larvae stage; the insect resumes development in a pupation phase. Adult WSS emerge from the previous year’s crop stubble in the spring(typically from May to June), and females lay their eggs inside wheat stems. Adult WSS flight takes place over 3-6 weeks[2], with individual adult insects living for about 7 days[12]. Eggs hatch, larvae feed on parenchyma tissues and bore through vascular bundles, then move to soil level to enter diapause, restarting their life cycle[12, 13]. WSS thrives in dry weather but requires adequate moisture for adult emergence[14, 15]. Temperature, and moisture significantly influence flight length and success[16]. Warmer conditions and calm winds enhance population growth by facilitating mating and oviposition[12].

Several pest control strategies are employed to reduce losses from WSS, but none by themselves have been shown to provide completely effective control[2]. Amongst different management strategies, cultural control methods[1, 6, 17, 18], biological control[19–23], and solid and semi-solid stem cultivars[24–32]have achieved varying success, but not fully effectively mitigated losses from WSS. WSS is extremely difficult to control with insecticides because the insects spend most of their lifecycle protected inside the stem as larvae, and managing adult populations has proved to be an ineffective way to reduce the population so far[1]. In addition, spraying insecticides during adult WSS emergence requires excessive monitoring and precise timing to have any effect, which may be difficult as the wasps can emerge over a 30-day period[33].

Due to the varying effectiveness of current WSS management strategies, growers and researchers continue to seek more reliable methods.One such strategy involves modeling the seasonal phenology of WSS from field monitoring data. Accumulated GDD have assisted farmers in various ways such as predicting insect emergence, and population peak, anticipating damage, determining optimal sampling times, and guiding appropriate management actions[34]. This approach has potential management implications, such as timing insecticide treatments to coincide with predictions of high pest pressure. Phenology models based on GDD have been successfully established and are very effective in supporting management strategies for insect pests[35–38].For example, the oviposition phenology model for grape berry moth helped reduce insecticide applications, increasing the efficacy of a single sprayin grape berries[39]. Similarly, using the GDD model instead of a calendar-based insecticide application for billbugs resulted in population reduction[40].

To date, several studies have examined the impact of weather on WSS phenology and ecology. Achhami et al. [41]found that GDD correlated with pre-diapause larval mortality in barley cultivars.Beres et al. [42]demonstrated that GDD was more effective than calendar days in estimating WSS population peaks in Montana wheat cultivars. In addition, Perez-Mendoza and Weaver [10]demonstrated that temperature and humidity affect WSS larval development and adult emergence, using data from three Montana WSS populations to identify optimal conditions for shorter developmental times and extreme conditions where development ceases.These complementary studies provide valuable resources that can potentially help mitigate WSS damage.Here, we use a long-term data set based on multiple years of adult WSS monitoring in Colorado to predict the seasonal phenology of WSS and determine the effects of temperature and precipitation on WSS populations in Colorado winter wheat.

# Methods

## Data collection

**Field sites.**Adult WSS monitoring was performed in winter wheat fields located at New Raymer and Orchard, CO (Table 1). These sites were chosen because WSS was first reported here in 2011. These sites were monitored every year from 2011 to 2023 except for 2015, when data was not collected due to adverse weather conditions, which prevented sampling. The data includes 6-9 sites per year; the variation in the number of fields sampled per year is attributed to factors such as crop rotation and/or low WSS population density. In Colorado, due to the lack of water, growers follow the wheat-fallow rotation, which consists of leaving a portion of the land without sowing for one crop season; this helps with retaining moisture for the next field season. Therefore, the fields sampled had little variation in their geographical location through the 13 years.

**Samples.**The sampling occurred on private land, and permission to conduct research was granted from each land owner of the sites. At each site, 100 180-degree sweeps were performed close to the edges of the fields using a canvas sweep net. Sampling would typically start by the middle or end of April and continue bi-weekly until mid-June. WSS are more active at higher temperatures,so sampling was conducted after 11 am during the warmest part of the day. WSS adult samples were placed in plastic zip-lock bags with their respective date and location and then brought back to the laboratory to be captured.

### Table 1. This is the Table 1 Title.Wheat fields sampled and their nearest corresponding locations.

| Field | Location | Latitude | Longitude |
| --- | --- | --- | --- |
| 1 | New Raymer, CO | 40.595973 | -103.898422 |
| 2 | New Raymer, CO | 40.591324 | -103.897172 |
| 3 | New Raymer, CO | 40.591277 | -103.898672 |
| 4 | New Raymer, CO | 40.587007 | -103.898172 |
| 5 | New Raymer, CO | 40.584113 | -103.896798 |
| 6 | New Raymer, CO | 40.582215 | -103.889426 |
| 7 | Orchard, CO | 40.468039 | -104.066917 |
| 8 | Orchard, CO | 40.482110 | -104.075203 |
| 9 | Orchard, CO | 40.474342 | -104.102564 |

**Temperature and precipitation.** We used CoAgMET(<https://coagmet.colostate.edu/>) to access temperature and precipitation data forthe past 12 years from weather stations near the field sites. Stations “New Raymer 21” and “Briggsdale” were chosen, both located within approximately 22 miles of the fields where the surveys were conducted. Minimum, mean, and maximum temperatures were retrieved for each day from January 1^st^ to December 31^st^ of each year and cumulative growing DD (GDDs) were generated with a biofix (specific date when monitoring of GDD begins for an insect) of January 1^st^, a base temperature of 10°C, and an upper horizontal cutoff threshold of 30°C. A base temperature of 10°C was chosen because it is commonly used in phenological models, and previous research has shown that roughly 90 days of development at 10°C are required to break the WSS obligatory larval diapause[11, 43].The upper-temperature threshold of 30°Cwas again selected because it is commonly used in phenological models, and previous research has indicated this is an optimal developmental temperature for WSS larvae[10]. A single sine method was used to generate DD from daily minimum and maximum temperatures, as this method has been demonstrated to better approximate true daily thermal accumulation from minimum and maximum temperatures than the traditional simple average growing degree-day method[44, 45].

## Statistical analysis

Two approaches were used to characterize the phenology of the spring WSS flight based on degree-day accumulation: a GLM/GAM approach using the methods previously described[46]. The GLM/GAM method allows for including other factors in the initial model construction before isolating the coefficients associated with the degree-day term and constructing a phenology model based on those coefficients, representing relative abundances at given degree-day accumulations. The GAM model fit to those isolated coefficients can be used to identify the start, peak, and end points of an insect emergence such as WSS in Colorado. However, this model is not easily used to characterize the degree-day accumulation associated with a specific fraction of the WSS flight (such as 10% or 90%), which would be valuable when communicating pest management recommendations to stakeholders. Transforming insect catches into a cumulative proportion of total captures and modelling the distribution with a probit function allows the model to predict degree-day accumulations associated with specific times in the WSS flight. Thelmer and mgcv package of R Version 2023.12.0 + 369was used for statistical analysis.

**Generalized additive mixed (GAM) phenology model.**Based on the methods detailed in Frost et al. 2013[47], we constructed a GAM model with cumulative DD, cumulative precipitation, site ID, and site-by-year interaction terms, all included as random effects, and WSS capture per 100 sweeps as the response variable to ultimately generate a phenological relationship between WSS captures and cumulative DD. A Poisson distribution was chosen for the model because it closely fits the distribution of observed WSS captures (full dataset, n=669). The inclusion of terms other than DD in the GAM model serves only to isolate the effect of DD on WSS captures from other possible influences. Next, the random effect coefficients for the DD term were collected from this full GAM model and used as the basis for generating a second GAM model that fits a smooth curve through these GDD coefficients. Points on this smooth fit curve were identified where the value crossed from negative to positive (start of WSS emergence), reached a maximum (peak emergence), and crossed back to negative (end of flight). The GAM model iswss_capture ~ 1|gdd + 1|precip + 1|location_year + 1|site_id, where each term is included as a random effect.

**Cumulative proportion generalized linear phenology model (GLM).** As an alternative statistical approach for characterizing the flight of WSS based on accumulated DD, we merged all years of WSS sweep captures, sorted the dataset by the cumulative DD associated with each capture, and transformed each WSS capture into a cumulative proportion of all WSS captures (cumulative capture divided by total capture). The GLM was then fit with the formula *wss_cumulative_proportion ~ cumulative_degree_days,* and a quasibinomial distribution was chosen with a probit link function.

**Environmental factors GAM model.** While both the GAM and GLM models provided insights into the timing of the flight, we lacked a framework for understanding environmental conditions either before or during the WSS flight that could affect the magnitude or severity of the emergence. To investigate these relationships, we constructed a GAM model that initially contained summary terms generated from the WSS dataset that were hypothesized to influence the magnitude of the WSS population. These terms included the day of the year of first detection, the number of days over which the flight took place, the mean GDD per day during the flight, the total snow accumulation during the prior winter, the total rainfall before the WSS flight, the incremental rainfall during the WSS flight, the minimum temperature recorded before or during the flight, the minimum and maximum temperature before or during the flight, and the minimum and maximum temperature during the flight period. In addition to these fixed terms, random effect smooth terms were added to the model to account for unexplained variance associated with the year and the field where sampling occurred. After fitting the full model, backward selection was used to incrementally drop the least significant terms from the model until only significant effects remained.

# Results

## Observed WSS phenology

The calendar date of emergence and peak WSS population were highly variable across years.WSS populations began to increase in early to mid-Mayand peaked in late May to early June except for 2012 (**Fig 1**).In 2012, unusually warm and dry winter and spring conditions led to much earlier WSS emergence, causing the population peak to occur up to 20 days earlier than usual (**Table S1**).This high degree of calendar variability in WSS emergence makes anticipating the flight very difficult. It underscores the need for a DD-based phenological model that models past flights effectively and provides a framework for predicting current-year flight initiation.

**Fig 1. This is the Fig 1 Title. Wheat stem sawfly (WSS) seasonal phenology from 2011 to 2023.** Smooth fit curves illustrate the average number of WSSadults caught per 100 sweepsby date and year. Depending on the year, the timing of the WSS flight relative to the calendar date is highly variable.

Using cumulative DD significantly improves overlap in observed WSS annual phenology curves across years relative to using simple calendar dates (**Fig 2**). While the magnitude of each year’s flight (e.g. total WSS captures) does vary, the timing of the flight's start, peak, and end points are much more closely aligned when using DD instead of calendar day. While there is still some remaining annual variability in phenology timing, and significant variation in total WSS captures per year, we attempt to explain these characteristics using some environmental data, explained later in the results.

**Fig 2. This is the Fig 2 Title.Relationship between log-normalized adult wheat stem sawfly in 100 sweeps made weekly and cumulative DD over the thirteen years of sampling.**Each individual year’s WSS flight is illustrated with colored smooth-fit lines (method: loess), while the combined dataset is modeledusing a GAM and shown in a bold red line. This model is the basis for generating the GAM degree-day model.

## WSS degree-day models

Using the GLM/GAM modeling approach, the start of the WSS flight was estimated to be 148cumulative Celsius growing DD (GDD, base 10°C, upper 30°C), the peak at 224 GDD, and the end at 354 GDD (**Fig. 3**). Based on actual WSS sweep net captures, we found that 4.7% (1,723) occurred before 148 GDD, 39.8% (14,496) before 224 GDD, 60.2% (21,957) after 224 GDD, and 0.4% (129) after 354 GDD. Expanding the peak to ±10 GDD (214–234 GDD), 23.3% (8,493) of captures occurred during this peak period (approximately 4 days at an average GDD accumulation of 5.4 per day). The equivalent phenology expressed in Fahrenheit growing DD (FDD) would use a base temperature of 50°F, an upper threshold of 86°F, starting at 266 FDD, peaking at 403 FDD, and ending at 637 FDD.

**Fig 3. This is the Fig 3 Title.GLM/GAM-derived WSS emergence phenology model**. Random effect coefficients associated with the cumulative growing degree-day term in the initial GLM are modeled using GAM. These coefficients represent relative over/under abundance of WSS captures after controlling for variance associated with year and location, and the points where the modeled fit crosses the axis or reaches a local maximum are used to identify the start, peak, and end of the WSS emergence period. Here, emergence is predicted to begin at 148 cumulative Celsius growing DD (GDD), peak at 224 GDD, and decline at 354 GDD, with a biofix of Jan 1.

Using a cumulative proportion probit fit, our second phenology modeling approach produced a similar result to the GLM/GAM approach. In addition, it allowed for the characterization of additional points along the WSS emergence distribution. The results of this model generally aligned with the previously described GAM model, though this GLM model predicted a slightly earlier initiation to the flight relative to the GAM. Using this curve, we find that 1% of WSS emerge by 125 GDD, 5% emerge by 154 GDD (roughly equivalent to the start point in the GLM/GAM model), 10% emerge by 169 GDD, 50% emerge by 223 GDD (peak emergence), 90% by 276 GDD, 95% by 291 GDD, and 99% by 320 GDD (**Fig. 4**). This model has an R^2^ of 0.987. The cumulative GDD term is highly significant (*t*=74.77, df=623, *P*<.0001).

**Fig 4. This is the Fig 4 Title.Probit fit of the cumulative proportion of total WSScaptures over cumulative growing DD**. Observed WSS captures (black points above) from 2011-2023 were arranged by cumulative growing degree-day (base 10°C, upper threshold 30°C, biofix Jan 1), and captures were converted into a cumulative proportion of all captures. As shown in red, a GLM with a quasibinomial distribution and a probit link function was used to characterize specific points along the modeled WSS emergence period.

## Environmental factors affecting WSS population size

Significant factors influencing mean WSS capture were the length of the emergence period (positive effect, *t*=2.03, *P*=0.046), the amount of rain accumulated between Jan 1 and the start of the WSS emergence (negative effect, *t*=-3.53, *P*=0.0007), the minimum temperature during the emergence period (positive effect, *t*=3.11, *P*=.003), the maximum temperature before WSS emergence (positive effect, *t*=2.21, *P*=0.03), and the maximum temperature during the emergence (negative effect, *t*=-5.72, *P*<0.0001). Factors that were not significant and were dropped from the reduced model included the day of year of first emergence, the mean daily GDD accumulation during the emergence period, the amount of snowfall during the prior winter, the amount of rainfall during the emergence period, and the minimum temperature before the start of emergence. The model also accounts for the random effects of the year (*F*=16.6,df=11,*P*<.0001) and site (*F*=9.68, df=13, *P*<.0001) using smooth terms. The reduced model has an R^2^of 0.744 and explains 79.7% of the variance in mean WSS captures between sites and years. See **Table 2** for a summary of significant model terms. These results suggest that higher WSS populations are associated with longer emergence periods, less precipitation before emergence, milder low temperatures during emergence, and milder maximum temperatures before and during emergence.

### Table 2. This is the Table 2 Title.GAM model results show environmental factors influencingmean wheat stem sawfly (WSS)captures.

| Term | Min | Max | Mean | Coefficient | Std. Error | t-value | Pr(>\|t\|) | Sig. |
| --- | --- | --- | --- | --- | --- | --- | --- | --- |
| (Intercept) |  |  |  | 7.283 | 4.453 | 1.64 | 0.106 |  |
| Emergence length (days) | 6 | 43 | 21 | 0.058 | 0.029 | 2.03 | 0.046 | * |
| Prior rainfall (mm) | 68.6 | 256.5 | 147.1 | -0.021 | 0.006 | -3.53 | 0.0007 | *** |
| Min temp during emergence (°C) | -1.7 | 6.7 | 4.5 | 0.348 | 0.112 | 3.11 | 0.0026 | ** |
| Max temp before emergence (°C) | 27.2 | 32.8 | 30.1 | 0.300 | 0.136 | 2.21 | 0.0299 | * |
| Max temp during emergence (°C) | 24.4 | 37.2 | 29.4 | -0.407 | 0.071 | -5.72 | <.0001 | *** |

# Discussion

Wheat stem sawfly (WSS) has long been considered the most important pest of wheat in the northern Great Plains of North America. In recent years, damage from WSS has expanded to areas of Nebraska, Wyoming, Colorado, and Kansas.With climate change, WSS populations are predicted to increase and expand to new geographical areas[15].The WSS has a univoltine life cycle and is a stem-boring insect where the larvae spend most of their time inside the stem and then undergo diapause in the wheat stubble [1,6], which makes the development of forecasting tools a challenge. Previous models attempted to predict WSS cutting[29] and found that stem cutting is influenced by precipitation. Other models generated data on larval mortality to provide information for future phenology models that can estimate WSS population density[48]. There is limited information on the phenology of WSS, especially in areas where infestations are more recent, such as Colorado. Here, we leveraged a thirteen-year dataset of adult WSS captures to predict the seasonal phenology of WSS and determine the effects of temperature and precipitation on WSS populations in Colorado. We found that WSS populations in Colorado wheat fields follow predictable patterns based on growing degree-day (GDD).The GLM model analyzed the weekly adult WSS captures over the thirteen-year period, which is useful for predicting field populations based on unique DD estimates. The model provided GDD approximations for WSS seasonal population trends across the thirteen-year period, with WSS first adult appearance predicted at 148 GDD, population peak at 224 GDD, and decline at 359 GDD. This represents the first published phenology model for WSS in Colorado.

When interpreting the data, it is recommended to start scouting for WSS before 148 GDD. Since spring GDD accumulation is variable, beginning scouting at 100 GDD is advisable to prepare for years like 2012, when emergence occurred 20 days earlier than average. From emergence until population peak, it typically takes around two weeks in Colorado. The average date of population peak across the 13-year period is May 28^th^ , which closely fits in the two-week average between emergence and peak for the WSS population. This average date concurs with previous research from McCullough et al. [49] that sampled winter wheat fields for two years in Nebraska and found the peak to happen end of May and beginning of June in the Central Great Plains. The second phenology modeling approach, using a cumulative proportion probit fit, produced results that were in alignment with the GLM/GAM approach but offered an enhanced characterization of WSS emergence distribution. The cumulative proportion probit fit model identified key emergence points, these points provide a more nuanced understanding of the WSS emergence pattern, offering critical data for early intervention and monitoring. The probit function confirms that the peak emergence at 223 GDD is consistent with the GLM/GAM model, validating the robustness of our model. The benefit of this model is that it can be used to predict the degree-day accumulation associated with any given fraction of the WSS flight, such as the 1% or 5% initial accumulation points, the 50% accumulation point (peak flight), and the end of the flight (95% or 99%). Understanding the distribution of emergence can help develop adaptive management strategies responsive to annual variations in weather conditions, particularly temperature. Also, identifying specific GDD emergence timing can aid in cultural control methods such as swathing timing or trap crop cutting timing to reduce the WSS population.The model also indicates when WSS populations are emerging in significant numbers. This information can optimize the timing of insecticide applications, enhancing their efficacy. Studies have shown that insecticides are often ineffective against WSS because the emergence window spans over a month, reducing exposure for adults emerging after spraying[50–52]. Therefore, this research suggests targeting insecticide applications during the initial population build-up.Further, males typically emerge first[10, 43], so phenology models such as the one presented here can be used to reduce the number of males before mating takes place. This strategy makes it possible to reduce female populations for the following year since WSS are haplodiploid, and unfertilized eggs will produce male offspring[43].

We found that the length of the WSS emergence period can positively impact WSS captures: a longer emergence period is associated with higher WSS captures, suggesting that an extended period allows for a larger portion of the population to emerge and survive.The amount of rainfall accumulated preceding WSS emergence negatively impacted WSS captures. This is in agreement with previous research that found that dry conditions preceding adult emergence could lead to higher WSS captures[14, 15]. In contrast, rainfall had a positive impact on larval survival with larval survival increasing with increasing weekly rainfall. Further, the minimum temperature during the emergence period positively impacted WSS captures. The maximum temperature before emergence can positively affectthe WSS population, whereas the maximum temperature during emergence has a negative effect.Previous research demonstrated that post-diapause larvae (preceding adult emergence) cannot complete development or will reenter diapause under low temperatures (< 10°C),andadult WSS may have high mortality under high temperatures(> 35°C) [9–11,53].Moreover, increasing temperatures preceding emergence potentially influencesthe suitability ofwinter wheat host plants.Overall, by incorporating 13 years of data from the same locations, we developed a robust GDD model to forecast WSS emergence and population growth, leading to more timely and effective management strategies.This model has been made availablefor Colorado wheat growers through theColoradoWheat website (<https://coloradowheat.org/>) and newsletters. Future research may consider interactions between GDD, precipitation, and WSS emergence across wheat cultivars in different locations, which will allow for more precise field population trend predictions and the development of preventative management options to control WSS.

**Acknowledgements**

We thank the members of the CSU insectary for collecting and processing the WSS samples, the Wickstrom and Mertens families for allowing us to use their fields, and the Franklin Graybill Statistical Laboratory at CSU for statistical consulting. Funding was provided by the Colorado Wheat Administrative Committee (CWAC) and the Colorado Sustainable Pest Management Center.

**References**

1.	Beres BL, Dosdall LM, Weaver DK, Cárcamo HA, Spaner DM (2011) Biology and integrated management of wheat stem sawfly and the need for continuing research. The Canadian Entomologist 143:105–125

2.	Irell B, Peairs F (2014) Wheat stem sawfly: A new pest of Colorado wheat. Colorado State University Extension, https://extension.colostate.edu/topic-areas/insects/wheat-stem-sawfly-a-new-pest-of-colorado-wheat-5-612/ (accessed on 4 April 2024).

3.	Macedo TB, Weaver DK, Peterson RKD (2007) Photosynthesis in wheat at the grain filling stage is altered by larval wheat stem sawfly (Hymenoptera: Cephidae) injury and reduced water availability. Journal of Entomological Science 42:228–238

4.	Holmes ND (1977) The effect of the wheat stem sawfly, *Cephus cinctus* (Hymenoptera: Cephidae), on the yield and quality of wheat. The Canadian Entomologist 109:1591–1598

5.	Morrill WL, Gabor JW, Hockett EA, Kushnak GD (1992) Wheat stem sawfly (Hymenoptera: Cephidae) resistance in winter wheat. Journal of Economic Entomology 85:2008–2011

6.	Ainslie CN (1920) The western grass-stem sawfly. Entomological Assistant, Cereal and Forage Insect Investigations 841

7.	Cockrell DM, Randolph T, Peirce E, Peairs FB (2021) Survey of wheat stem sawfly (Hymenoptera: Cephidae) infesting wheat in eastern Colorado. Journal of Economic Entomology 114:998–1004

8.	Wasserman-Olin R, Hill R, Osterholzer A, Nachappa P (2024) The economic impact of wheat stem sawfly on Colorado wheat. Colorado State University Extension 47-48, https://webdoc.agsci.colostate.edu/csucrops/reports/winterwheat/wheatreport_2024_WFD.pdf (accessed on 6 September 2024).

9.	Villacorta A, Bell RA, Callenbach JA (1972) Respiratory activity during development and diapause of *Cephus cinctus* (Hymenoptera: Cephidae), with emphasis on effect of temperature. Annals of the Entomological Society of America 65:419–422

10.	Perez-Mendoza J, Weaver DK (2006) Temperature and relative humidity effects on postdiapause larval development and adult emergence in three populations of wheat stem sawfly (Hymenoptera: Cephidae). Environmental Entomology 35:1222–1231

11.	Salt R (1947) Some effects of temperature on the production and elimination of diapause in the wheat stem sawfly, *Cephus cinctus* Nort. Canadian Journal of Research 25:66–86

12.	Wallace L, McNeal F (1966) Stem sawflies of economic importance in grain crops in the United States. Entomology Research Division

13.	Macedo TB, Peterson RKD, Weaver DK, Morrill WL (2005) Wheat stem sawfly, *Cephus cinctus* Norton, impact on wheat primary metabolism: an ecophysiological approach. Environmental Entomology 34:719–726

14.	Criddle N (1923) Popular and practical entomology: the life habits of *Cephus cinctus* Nort. in Manitoba. The Canadian Entomologist.

15.	Olfert O, Weiss RM, Catton H, Cárcamo H, Meers S (2019) Bioclimatic assessment of abiotic factors affecting relative abundance and distribution of wheat stem sawfly (Hymenoptera: Cephidae) in western Canada. The Canadian Entomologist 151:16–33

16.	Seamans HL (1945) A preliminary report on the climatology of the wheat stem sawfly (*Cephus cinctus* Norton.) on the canadian prairies. Scientific Agriculture 25:432–457

17.	Lafond GP, Boyetchko SM, Brandt SA, Clayton GW, Entz MH (1996) Influence of changing tillage practices on crop production. Canadian Journal of Plant Science 76:641–649

18.	Lal R (1997) Residue management, conservation tillage and soil restoration for mitigating greenhouse effect by CO2-enrichment. Soil & Tillage Research 43:81–107

19.	Nelson WA, Farstad CW (1953) Biology of *Bracon cephi* (Gahan) (Hymenoptera: Braconidae), an important native parasite of the wheat stem sawfly, *Cephus cinctus* Nort. (Hymenoptera: Cephidae), in western Canada. The Canadian Entomologist 85:103–107

20.	Runyon JB, Morrill WL, Weaver DK, Miller PR (2002) Parasitism of the wheat stem sawfly (Hymenoptera: Cephidae) by *Bracon cephi* and *B. lissogaster* (Hymenoptera: Braconidae) in wheat fields bordering tilled and untilled fallow in Montana. Journal of Economic Entomology 95:1130–1134

21.	Buteler M, Weaver DK, Miller PR (2008) Wheat stem sawfly-infested plants benefit from parasitism of the herbivorous larvae. Agricultural and Forest Entomology 10:347–354

22.	Peterson RKD, Buteler M, Weaver DK, Macedo TB, Sun Z, Perez OG, Pallipparambil GR (2011) Parasitism and the demography of wheat stem sawfly larvae, *Cephus cinctus*. BioControl 56:831–839

23.	Buteler M, Peterson RKD, Hofland ML, Weaver DK (2015) A multiple decrement life table reveals that host plant resistance and parasitism are major causes of mortality for the wheat stem sawfly. Environmental Entomology 44:1571–1580

24.	Eckroth EG, McNeal FH (1953) Association of plant characters in spring wheat with resistance to the wheat stem sawfly. Agronomy Journal 45:400–404

25.	Holmes ND (1984) The effect of light on the resistance of hard red spring wheats to the wheat stem sawfly, *Cephus cinctus* (Hymenoptera: Cephidae). The Canadian Entomologist 116:677–684

26.	Delaney KJ, Weaver DK, Peterson RKD (2010) Photosynthesis and yield reductions from wheat stem sawfly (Hymenoptera: Cephidae): Interactions with wheat solidness, water stress, and phosphorus deficiency. Journal of Economic Entomology 103:516–524

27.	Beres BL, McKenzie RH, Cárcamo HA, Dosdall LM, Evenden ML, Yang RC, Spaner DM (2012) Influence of seeding rate, nitrogen management, and micronutrient blend applications on pith expression in solid-stemmed spring wheat. Crop Science 52:1316–1329

28.	Nilsen KT, Clarke JM, Beres BL, Pozniak CJ (2016) Sowing density and cultivar effects on pith expression in solid-stemmed durum wheat. Agronomy Journal 108:219–228

29.	Beres BL, Hill BD, Carcamo HA, Knodel JJ, Weaver DK, Cuthbert RD (2017) An artificial neural network model to predict wheat stem sawfly cutting in solid-stemmed wheat cultivars. Canadian Journal of Plant Science 97:329–336

30.	Subedi M, Cárcamo HA, Knodel JJ, Weaver DK, Cuthbert RD, Pozniak CJ, Nilsen KT, Beres BL (2021) Stability analysis of stem solidness, grain yield, and grain protein concentration in spring wheat. Canadian Journal of Plant Science 101:456–475

31.	Bathini A, Mendu L, Singh NP, Cook J, Weaver D, Sherman J, Hager M, Mondal S, Mendu V (2023) A “solid” solution for wheat stem sawfly (Hymenoptera: Cephidae) resistance: Genetics, breeding and development of solid stem wheat. Crop Science 63:1934–1948

32.	Peirce ES, Evers B, Winn ZJ, et al (2024) Identifying novel sources of resistance to wheat stem sawfly in five wild wheat species. Pest Management Science.

33.	Knodel JJ, Beauzay PB, Eriksmoen ED, Pederson JD (2009) Pest management of wheat stem maggot (Diptera: Chloropidae) and wheat stem sawfly (Hymenoptera: Cephidae) using insecticides in spring wheat. Journal of Agriculture and Urban Entomology 26:183–197

34.	Leybourne DJ, Storer KE, Marshall A, Musa N, Telling S, Abel L, White S, Ellis S, Yang P, Berry PM (2024) Thresholds and prediction models to support the sustainable management of herbivorous insects in wheat. A review. Agronomy for Sustainable Development.

35.	Teixeira LAF, Polavarapu S (2001) Postdiapause development and prediction of emergence of female blueberry maggot (Diptera: Tephritidae). Environmental Entomology 30:925–931

36.	Frost KE, Esker PD, Van Haren R, Kotolski L, Groves RL (2013) Seasonal patterns of aster leafhopper (Hemiptera: Cicadellidae) abundance and aster yellows phytoplasma infectivity in Wisconsin carrot fields. Environmental Entomology 42:491–502

37.	Kamiyama MT, Bradford BZ, Groves RL, Guédot C (2020) Degree day models to forecast the seasonal phenology of *Drosophila suzukii* in tart cherry orchards in the Midwest U.S. PLoS ONE.

38.	Ebbenga DN, Hanson AA, Burkness EC, Hutchison WD (2022) A degree-day model for forecasting adult phenology of *Popillia japonica* (Coleoptera: Scarabaeidae) in a temperate climate. Frontiers in Insect Science.

39.	Teixeira LAF, Mason K, Isaacs R (2009) Control of grape berry moth (Lepidoptera: Tortricidae) in relation to oviposition phenology. Journal of Economic Entomology 102:692–698

40.	Van Dyke A, Wickwar D, Dupuy MM, Ramirez RA (2023) Evaluating insecticide application timings against billbugs (*Sphenophorus spp*.) using a degree-day model and calendar-based approach in the Intermountain West. Crop, Forage and Turfgrass Management.

41.	Achhami BB, Reddy GVP, Sherman JD, Peterson RKD, Weaver DK (2020) Effect of precipitation and temperature on larval survival of *Cephus cinctus* (Hymenoptera: Cephidae) in barley cultivars. Journal of Economic Entomology 113:1982–1989

42.	Beres BL, Cárcamo HA, Dosdall LM, Yang RC, Evenden ML, Spaner DM (2011) Do interactions between residue management and direct seeding system affect wheat stem sawfly and grain yield? Agronomy Journal 103:1635–1644

43.	Holmes N (1979) The wheat stem sawfly. 26th Annual Meetings of the Entomological Society of Alberta and Entomological Society of Saskatchewan 26

44.	Allen JC (1976) A modified sine wave method for calculating degree days. Environmental Entomology 5:388–396

45.	Pruess KP (1983) Day-Degree methods for pest management. Environmental Entomology 12:613–619

46.	Jagemann SM, Juzwik J, Tobin PC, Raffa KF (2018) Seasonal and regional distributions, degree-day models, and phoresy rates of the major sap beetle (Coleoptera: Nitidulidae) vectors of the oak wilt fungus, *Bretziella fagacearum*, in Wisconsin. Environmental Entomology 47:1152–1164

47.	Frost KE, Esker PD, Haren RV, Kotolski L, Groves RL (2013) Factors influencing aster leafhopper (Hemiptera: Cicadellidae) abundance and aster yellows phytoplasma infectivity in Wisconsin carrot fields. Environmental Entomology 42:477–490

48.	Sjolie DM, Willenborg CJ, Vankosky MA (2024) The causes of wheat stem sawfly (Hymenoptera: Cephidae) larval mortality in the Canadian prairies. The Canadian Entomologist.

49.	McCullough CT, Hein GL, Bradshaw JD (2020) Phenology and dispersal of the wheat stem sawfly (Hymenoptera: Cephidae) into winter wheat fields in Nebraska. Journal of Economic Entomology 113:1831–1838

50.	Wallace LE (1962) Field-plot tests of chemicals for wheat stem sawfly control. Journal of Economic Entomology 55:908–912

51.	Holmes ND, Peterson LK (1963) Heptachlor as a systemic insecticide against the wheat stem sawfly, *Cephus cinctus* Nort. The Canadian Entomologist 95:792–796

52.	Wallace LE, Butler LI (1967) Residues of heptachlor and heptachlor epoxide in wheat treated to control the wheat stem sawfly. Journal of Economic Entomology 60:254–256

53.	Lou KF, Weiss MJ, Bruckner PL, Morrill WL, Talbert LE, Martin JM (1998) RAPD variation within and among geographic populations of wheat stem sawfly (*Cephus cinctus* Norton). The American Genetic Association 89:329–335
