# Supplementary material for: A new growing degree-day phenology model for wheat stem sawfly (Hymenoptera: Cephidae) in Colorado wheat fields
Source: PLoS One. 2025 Apr 7;20(4):e0320497. doi: 10.1371/journal.pone.0320497 (PMC11975124; doi:10.1371/journal.pone.0320497)
Supplement: Attachment — Submitted filename: Research evaluation 11.docx [file pone.0320497.s003.docx]

**Research evaluation**

**A new degree-day phenology model for wheat stem sawfly (Hymenoptera: Cephidae) in Colorado wheat fields**

**Research Summary**

The idea of the research is concerned with an important topic in the field of Modeling of the Wheat stem sawfly (WSS), *Cephus cinctus* Norton (Hymenoptera: Cephidae), to a phenology model that predicts adult WSS emergence and population peak based on growing DD (GDD). A better understanding of WSS emergence, population size, and related environmental factors is critical to building efficient and effective integrated pest management (IPM) strategies for this pest. Where wheat fields were sampled for adult WSS from mid-April to the end of June between 2011 and 2023 in several field sites in two locations in Colorado.

The inter-annual variability in emergence timing based on calendar date was substantially reduced when using a GDD model with a base temperature of 10°C, an upper threshold of 30°C, and a biofix of Jan 1. The model predicted initial WSS emergence at 148 DD, population peak at 224 DD, and decline at 354 DD. There also modeled the effects of environmental factors on mean WSS populations at each field site, finding that higher WSS populations are associated with longer emergence periods, less precipitation before emergence, milder temperatures during emergence, and milder maximum temperatures before and during emergence. By analyzing multiple years of comprehensive phenology data, they provide robust models to guide adult WSS forecasting and monitoring for the first time.

Further, this data will aid in decision-making related to timely and effective management strategies to suppress populations of WSS.

**The conclusion** is good. It shows the importance of the study in terms of the suitable by analyzing multiple years of comprehensive phenology data, there provided robust models to guide adult WSS forecasting and monitoring for the first time. Further, this data will aid in decision-making related to timely and effective management strategies to suppress populations of WSS, and it is complete and clear.

**Keywords** are not provided in the research.

**The introduction** is comprehensive and it deals with the subject of the study with a high professionalism and presents all the ideas related to the topic presented in the appropriate literature, where it referred to impact of weather on WSS phenology and ecology. Achhami et al. found that GDD correlated with pre-diapause larval mortality in barley cultivars. Beres et al. demonstrated that GDD was more effective than calendar days in estimating WSS population peaks in Montana wheat cultivars.

**The researchers pointed out the main problem** modeling the seasonal phenology of WSS from field monitoring data.

**In addition,** this approach has potential management implications, such as timing insecticide treatments to coincide with predictions of high pest pressure. Phenology models based on GDD have been successfully established and are very effective in supporting management strategies for insect pests.

To date, several studies have examined the impact of weather on WSS phenology and ecology. Achhami et al. found that GDD correlated with pre-diapause larval mortality in barley cultivars. Beres et al. demonstrated that GDD was more effective than calendar days in estimating WSS population peaks in Montana wheat cultivars. In addition, Perez-Mendoza and Weaver demonstrated that temperature and humidity affect WSS larval development and adult emergence, using data from three Montana WSS populations to identify optimal conditions for shorter developmental times and extreme conditions where development ceases. These complementary studies provide valuable resources that can potentially help mitigate WSS damage. Here, we use a long-term data set based on multiple years of adult WSS monitoring in Colorado to predict the seasonal phenology of WSS and determine the effects of temperature and precipitation on WSS populations in Colorado winter wheat.

**In this study,** Accumulated GDD have assisted farmers in various ways such as predicting insect emergence, and population peak, anticipating damage, determining optimal sampling times, and guiding appropriate management actions. This approach has potential management implications, such as timing insecticide treatments to coincide with predictions of high pest pressure. Phenology models based on GDD have been successfully established and are very effective in supporting management strategies for insect pests.

**Materials and methods** Sample collection was good and clear, terms included Adult WSS monitoring was performed in winter wheat fields located at New Raymer and Orchard, CO. These sites were chosen because WSS was first reported here in 2011. These sites were monitored every year from 2011 to 2023 except for 2015, when data was not collected due to adverse weather conditions, which prevented sampling. The data includes 6-9 sites per year; the variation in the number of fields sampled per year is attributed to factors such as crop rotation and/or low WSS population density. In Colorado, due to the lack of water, growers follow the wheat-fallow rotation, which consists of leaving a portion of the land without sowing for one crop season; this helps with retaining moisture for the next field season. Therefore, the fields sampled had little variation in their geographical location through the 13 years.

The sampling occurred on private land. At each site, 100 180-degree sweeps were performed close to the edges of the fields using a canvas sweep net. Sampling would typically start by the middle or end of April and continue bi-weekly until mid-June. WSS are more active at higher temperatures, so sampling was conducted after 11 am during the warmest part of the day. WSS adult samples were placed in plastic zip-lock bags with their respective date and location and then brought back to the laboratory to be captured.

**Statistical analysis**: Two approaches were used to characterize the phenology of the spring WSS flight based on degree-day accumulation: a GLM/GAM approach using the methods previously described. The GLM/GAM method allows for including other factors in the initial model construction before isolating the coefficients associated with the degree-day term and constructing a phenology model based on those coefficients, representing relative abundances at given degree-day accumulations. Generalized additive mixed (GAM) phenology model. Cumulative proportion generalized linear phenology model (GLM). Environmental factors GAM model.

**The results were well presented and consistent with the method of work, including the following main headings:** Observed WSS phenology, WSS degree-day models, Environmental factors affecting WSS population size, the results were good in the presentation method.

Using cumulative DD significantly improves overlap in observed WSS annual phenology curves across years relative to using simple calendar dates. While the magnitude of each year’s flight (e.g., total WSS captures) does vary, the timing of the flight's start, peak, and end points are much more closely aligned when using DD instead of calendar day. While there is still some remaining annual variability in phenology timing, and significant variation in total WSS captures per year, we attempt to explain these characteristics using some environmental data, explained later in the results.

Using the GLM/GAM modeling approach, the start of the WSS flight was estimated to be 148 cumulative Celsius growing DD (GDD, base 10°C, upper 30°C), the peak at 224 GDD, and the end at 354 GDD. Based on actual WSS sweep net captures, we found that 4.7% (1,723) occurred before 148 GDD, 39.8% (14,496) before 224 GDD, 60.2% (21,957) after 224 GDD, and 0.4% (129) after 354 GDD. Expanding the peak to ±10 GDD (214–234 GDD), 23.3% (8,493) of captures occurred during this peak period (approximately 4 days at an average GDD accumulation of 5.4 per day).

**Discussion paragraph**, Wheat stem sawfly (WSS) has long been considered the most important pest of wheat in the northern Great Plains of North America. In recent years, damage from WSS has expanded to areas of Nebraska, Wyoming, Colorado, and Kansas. With climate change, WSS populations are predicted to increase and expand to new geographical areas.

There is limited information on the phenology of WSS, especially in areas where infestations are more recent, such as Colorado. Here, they leveraged a thirteen-year dataset of adult WSS captures to predict the seasonal phenology of WSS and determine the effects of temperature and precipitation on WSS populations in Colorado. They found that WSS populations in Colorado wheat fields follow predictable patterns based on growing degree-day (GDD). The GLM model analyzed the weekly adult WSS captures over the thirteen-year period, which is useful for predicting field populations based on unique DD estimates. The model provided GDD approximations for WSS seasonal population trends across the thirteen-year period, with WSS first adult appearance predicted at 148 GDD, population peak at 224 GDD, and decline at 359 GDD. This represents the first published phenology model for WSS in Colorado.

They found that the length of the WSS emergence period can positively impact WSS captures: a longer emergence period is associated with higher WSS captures, suggesting that an extended period allows for a larger portion of the population to emerge and survive. The amount of rainfall accumulated preceding WSS emergence negatively impacted WSS captures. This is in agreement with previous research that found that dry conditions preceding adult emergence could lead to higher WSS captures. In contrast, rainfall had a positive impact on larval survival with larval survival increasing with increasing weekly rainfall.

Further, the minimum temperature during the emergence period positively impacted WSS captures. The maximum temperature before emergence can positively affect the WSS population, whereas the maximum temperature during emergence has a negative effect. Previous research demonstrated that post-diapause larvae (preceding adult emergence) cannot complete development or will reenter diapause under low temperatures (< 10°C), and adult WSS may have high mortality under high temperatures (> 35°C). Moreover, increasing temperatures preceding emergence potentially influences the suitability of winter wheat host plants. Overall, by incorporating 13 years of data from the same locations, we developed a robust GDD model to forecast WSS emergence and population growth, leading to more timely and effective management strategies.

**References** Well Attributed, Latest Reference 2024, I Found some minor errors in the list of references, 12, 14, 32, 34, 37, 38, 40, 43, 48 documentation needs to be completed.

**In conclusion**, I recommend publishing this research in your important and discreet journal due to its scientific importance, after making the required modifications.

**Latakia, 22/10/2024**

**Reviewer**

**Prof. Dr. Nabil ABO KAF**
